# Toxic Effect of a Marine Bacterium on Aquatic Organisms and Its Algicidal Substances against *Phaeocystis globosa*

**DOI:** 10.1371/journal.pone.0114933

**Published:** 2015-02-03

**Authors:** Qiuchan Yang, Lina Chen, Xiaoli Hu, Ling Zhao, Pinghe Yin, Qiang Li

**Affiliations:** 1 Key Laboratory of Water/Soil Toxic Pollutants Control and Bioremediation of Guangdong Higher Education Institutes, School of Environment, Jinan University, Guangzhou 510632, China; 2 Department of Chemistry, Jinan University, Guangzhou 510632, China; University of California, Merced, UNITED STATES

## Abstract

Harmful algal blooms have caused enormous damage to the marine ecosystem and the coastal economy in China. In this paper, a bacterial strain B1, which had strong algicidal activity against *Phaeocystis globosa*, was isolated from the coastal waters of Zhuhai in China. The strain B1 was identified as *Bacillus* sp. on the basis of 16S rDNA gene sequence and morphological characteristics. To evaluate the ecological safety of the algicidal substances produced by strain B1, their toxic effects on marine organisms were tested. Results showed that there were no adverse effects observed in the growth of *Chlorella vulgaris*, *Chaetoceros muelleri*, and *Isochrystis galbana* after exposure to the algicidal substances at a concentration of 1.0% (v/v) for 96 h. The 48h LC_50_ values for *Brachionus plicatilis*, *Moina mongolica* Daday and *Paralichthys olivaceus* were 5.7, 9.0 and 12.1% (v/v), respectively. Subsequently, the algicidal substances from strain B1 culture were isolated and purified by silica gel column, Sephadex G-15 column and high-performance liquid chromatography. Based on quadrupole time-of-flight mass spectrometry and PeakView Software, the purified substances were identified as prolyl-methionine and hypoxanthine. Algicidal mechanism indicated that prolyl-methionine and hypoxanthine inhibited the growth of *P. globosa* by disrupting the antioxidant systems. In the acute toxicity assessment using *M. mongolica*, 24h LC_50_ values of prolyl-methionine and hypoxanthine were 7.0 and 13.8 g/L, respectively. The active substances produced by strain B1 can be considered as ecologically and environmentally biological agents for controlling harmful algal blooms.

## Introduction

In recent years, harmful algal blooms (HABs) have become a significant problem in coastal regions worldwide due to their production of endogenous toxin, sheer biomass and physical shape [1–3]. To manage and mitigate the adverse impact of HABs, various management strategies including physical, chemical and biological methods have been developed. However, although physical methods can effectively control HABs within a short period after application, they are generally used as assistant methods because of the high cost and unsuitability for a large scale of operation. Chemical methods are easy to operate and can efficiently control the algal blooms, but they can cause serious secondary pollution. Therefore, economical and biological methods such as algicidal bacteria are considered as important tools to control HABs [4–6].

Recently, several strains of algicidal bacteria have been isolated from natural ecosystem, such as *Flavobacterium* sp. [7], *Vibrio* sp. [8], *Streptomycete* sp. [9], *Pseudoalteromonas* sp. [10] and *Actinomyces* sp. [6]. These algicidal bacteria inhibit the growth of harmful algae by attacking the cells either directly via cell-to-cell contact or indirectly via the release of algicdal substances [11, 12], including proteases [13], peptides [14], bio-surfactants [15, 16], amino acids [17] and antibiotics [18]. However, few purified compounds have been identified.

To ensure that the natural environment is not severely disturbed, the ecological response of aquatic organisms must be considered if the algicidal substances are to be developed as biocontrol agents [19]. Some algicidal substances do not specifically target harmful algae in aquatic environment, therefore using such algicidal substances may cause the collapse of aquatic ecological system owing to inhibition or lethal effects on non-target organisms. Luo et al. [6] indicated algicidal subatances produced by *Streptomyces* were not exclusive to *Microcystis aeruginosa* (algicidal rate was 62.7%), but also inhibited the growth of *Oscillatoria animalis* (algicidal rate was 62.9%) at the same concentration of 10% (v/v) after 6 d. Cho [12] indicated that four algicidal compounds produced by *Alteromonas* sp. KNS-16 showed ranges of 409–608 and 189–224 μg/mL of 48h LC_50_ for *Artemia salina* and *Brachionus rotundiformis*, respectively. Although some algicidal substances produced by bacteria have been isolated and their inhibitory effects on HABs have been tested, information on their toxicology relative to other aquatic organisms is very limited.

*Phaeocystis globosa* (*P. globosa*) is a notorious HAB species and frequently breaks out in China [20–22]. Although finding suitable biological algicides is of particular interest, few works of algicidal bacteria against *P. globosa* have been reported. In this study, an algicidal bacterium named B1 was isolated from the blooms of *P. globosa* in Zhuhai, China and identified as *Bacillus* sp. based on 16S rDNA gene sequence and morphological characteristics. The toxicities of algicidal substances produced by strain B1 against phytoplankton (*Chlorella vulgaris* (*C. vulgaris*), *Chaetoceros Muelleri* (*C. muelleri*), and *Isochrystis galbana* (*I. galbana*)), zooplankton (*Brachionus plicatilis* (*B. plicatilis*) and *Moina mongolica* Daday (*M. mongolica*)) and *Paralichthys olivaceus* (*P. olivaceus*) were evaluated. The algicidal substances were isolated and purified by silica gel column chromatography, Sephadex G-15 column chromatography and HPLC. The chemical structures of purified compounds from HPLC were further defined using quadrupole time-of-flight mass spectrometry (Q-TOF-MS) and PeakView software. The algicidal mechanisms to *P. globosa* cells and acute toxicities against *M. mongolica* of the purified compounds were also studied.

## Materials and Methods

### Ethics Statement

No specific permissions were required for the water sampling from the coast of Zhuhai (22°17′–22°20′N, 113°34′–113°36′E) in this study. The field study did not involve endangered or protected species. All procedures and animal studies were in accordance with the guideline approved by Chinese Association for Laboratory Animal Sciences.

### Test Organisms and Culture Conditions

All algae and zooplankton were obtained from the Research Center for Harmful Algae and Aquatic Environment, Jinan University. The species of algae, *P. globosa* (prymnesiophyte), *C. vulgaris* (green algae), *I. galbana* (chrysophyte) and *C. muelleri* (diatom) were cultivated in f/2 medium at 20 ± 0.5°C under a 12 h light/12 h dark cycle with a light intensity of 50 μmol photons m^−2^ s^−1^. The f/2 medium contained 75 mg NaNO_3_, 5 mg NaH_2_PO_4_·H_2_O, 3.15 mg FeCl_3_·6H_2_O, 4.36 g Na_2_EDTA·2H_2_O, 0.01 mg CuSO_4_·5H_2_O, 0.006 mg Na_2_MoO_4_, 0.02 mg ZnSO_4_, 0.01 mg CoCl_2_·6H_2_O, 0.18 mg MnCl_2_·4H_2_O, 0.10 mg Thiamine·HCl, 0.50 μg biotin and 0.50 μg Vitamin B_12_ per liter of filtered seawater. Zooplankton of *B. plicatilis* (rotifer) and *M. mongolica* (crustacean) were reared for 1–2 d in the laboratory at 20 ± 0.5°C. *P. olivaceus* (osteichthyes, body length 2.0 ± 1.0 cm) were obtained from Fish Hatchery in Zhuhai and maintained in artificial seawater at 20 ± 0.5°C for 5 days.

### Isolation and Screening of Algicidal Bacteria

Surface water samples in the blooms of *P. globosa* were collected from the coast in Zhuhai, China and serially diluted (10^−1^–10^−7^ fold) with sterilized seawater. An aliquot of each dilution (0.1 mL) was spread onto 2216E agar plate (peptone 5 g, yeast extraction 1 g, ferric phosphorous acid 0.1 g, agar 15 g, pH 7.6–7.8, fixed capacity to 1 L seawater) and incubated at 30°C for 3 d. Individual clones were picked from the plates and streaked onto 2216E agar to obtain purified isolates. Isolates were grown in 100 mL of 2216E liquid medium at 30°C for 3 d. A 1.0 mL aliquot of each isolate culture was inoculated in triplicate into 100 mL of logarithmic-phase *P. globosa* cultures, and a 1.0 mL aliquot of 2216E liquid medium was added to the algal cultures as a control. After incubation for 5 d, algicidal rate was tested by counting the algal cell number. Strain that showed the highest algicidal rate was further analyzed.

### Morphology and 16S rDNA Gene Identification of the Isolated Bacterium

Morphological observation of the strain B1 was carried out both with the naked eye and using a scanning electron microscopy (SEM, Philips XL-30ESEM, Holland) after cultivation on 2216E plates for 3 d at 30°C. Bacterial cells from strain B1 cultures grown in 2216E liquid cultures were collected by centrifugation (4000 × g for 10 min). The total DNA was extracted using a DNA extraction kit according to the manufacturer’s instructions (Genetech, China). Subsequently, the 16S rDNA was amplified by PCR using primers 27F (5’-GAGAGTTTGATCCTGGCTCAG-3’) and 1495R (5’-CTACGGCTACCTTGTTAC-GA-3’). The thermal profile was consisted of 35 cycles of denaturation at 94°C for 35 s, annealing at 55°C for 35 s, and an extension at 72°C for 90 s, followed by a final extension step at 72°C for 10 min. The PCR products were purified from agarose gel with GeneClean Turbo Kit (Qbiogene) and cloned into pMD 19-T vector following by sequencing which was performed by Shanghai Genetech Biotechnological Company. The nucleotide sequences were edited using the software Seaview [23]. A comparison of nucleotide sequences was performed using the BLASTN database (http://www.ncbi.nlm.nih.gov/BLAST) at the National Center for Biotechnology Information (NCBI). The sequence was aligned using the program ClustalW, and a phylogenetic tree was constructed using the neighbor-joining method in MEGA 4.0 software [24].

### Determination of Algicidal Activity

The algicidal activities of algicidal bacterium and its algicidal substances were determined by counting algal cells using a light microscope with a hemocytometer. The algal cells were counted after fixing a 5 mL aliquot of the culture with Lugol’s iodine. The algicidal rate could be calculated by the following formula:

$$\mathrm{Algicidal rate}(\%)=\frac{D_{c}-D_{t}}{D_{c}}\times 100$$

D_c_ and D_t_ represent the cell densities of algae in control and treatment group, respectively.

### Toxicity of Bacterial Supernatant on Algae, Zooplankton and Fish

Strain B1 was in standing 2216E liquid medium at 30°C with shaking at 160 rpm for 3 d and centrifuged at 4000 × *g* for 10 min to collect the supernatant and then incubated with *C. vulgaris, I. galbana* and *C. muelleri*, respectively. The cell density of algae was counted after 1, 2, 3 and 4 d. Algae were cultured in different concentrations of bacterial supernatant (0–2.5%, v/v) in triplicates. For acute toxicity to zooplankton and fish, ten samples of *B. plicatilis* or *M. mongolica* were randomly selected and introduced into a 50 mL glass beaker containing 20 mL of seawater, and ten *P. olivaceus* were added to 1 L of seawater. All acute toxicities tests were conducted without feeding during testing. The concentration of bacterial supernatant for zooplankton and fish were 1–64% and 4–32% (v/v), respectively. Control was conducted in seawater without bacterial supernatant. Both control and treatment were carried out in triplicates and mortality was recorded after 24 and 48 h.

### Isolation and Identification of Algicidal Substances

Strain B1 was cultured in 2216E liquid medium (4 L) at 30°C with shaking at 160 rpm for 3 d. The bacterial culture was centrifuged at 4000 × *g* for 10 min to collect the supernatant, and then filtered through a 0.22 μm microporous membrane. The filtrate was evaporated to dryness by using a rotary evaporator. The residue was applied to the silica gel column chromatography (the silica particle size: 200–300 mesh) and eluted with six volume ratios of CHCl_3_/CH_3_OH (5:5, 4:6, 3:7, 2:8, 1:9 and 0:1). Fractions that exhibited algicidal activity were subjected to Sephadex G-15 (Pharmacia) column chromatography with ultrapure water as eluent. The active fraction was applied to a reversed-phase C_18_ column (4.6 × 250 mm, Agilent) connected to an HPLC system and monitored at 254 nm using UV detector. The purified compounds from HPLC were identified by Q-TOF-MS and PeakView software (AB SCIEX TripleTOF 5600 + System with Accelerator TOF Analyzer and Electrospray Ionization source).

### Determination of Superoxide Dismutase, Catalase Activity and Malondialdehyde in *P. globosa*

Prolyl-methionine and hypoxanthine were purchased commercially and separately added to exponential-phase culture of *P. globosa* at an initial concentration of 20 μg/mL. Control was conducted in culture of *P. globosa* without prolyl-methionine and hypoxanthine. The cells of *P. globosa* were collected by centrifugation at 4000 × *g* for 10 min followed by rinsing in 1 mL of phosphate buffer saline (PBS, pH 7.8) and ultrasonicated at 400 W for 5 min. Cellular debris was removed by centrifugation at 8000 × *g* for 10 min at 4°C, and the supernatant was used to analyze Superoxide Dismutase (SOD), Catalase Activity (CAT) and Malondialdehyde (MDA). SOD activity was measured by nitroblue terazolium (NBT) photoreduction. CAT activity was determined by ultraviolet absorption method [25, 26]. The MDA was determined following the thiobarbituric acid method [27, 28].

### Statistical Analysis

All data were expressed as means ± standard deviation. The different means observed were determined by one-way analysis of variance and compared using Duncan’s test at a significance level of P < 0.05 and P < 0.01 using SPSS (version 10.1). The LC_50_ value for each exposure time was calculated from probit regression generated by SPSS 10.1.

## Results

### Screening and Identification of Algicidal Bacteria

A total of 17 bacterial strains were isolated from surface water samples which collected from the coast in Zhuhai, China, four of which were observed to have high algicidal activity (>75%) toward *P. globosa*, and strain B1 exhibited the strongest algicidal activity (94.3%).

**Figure 1:**
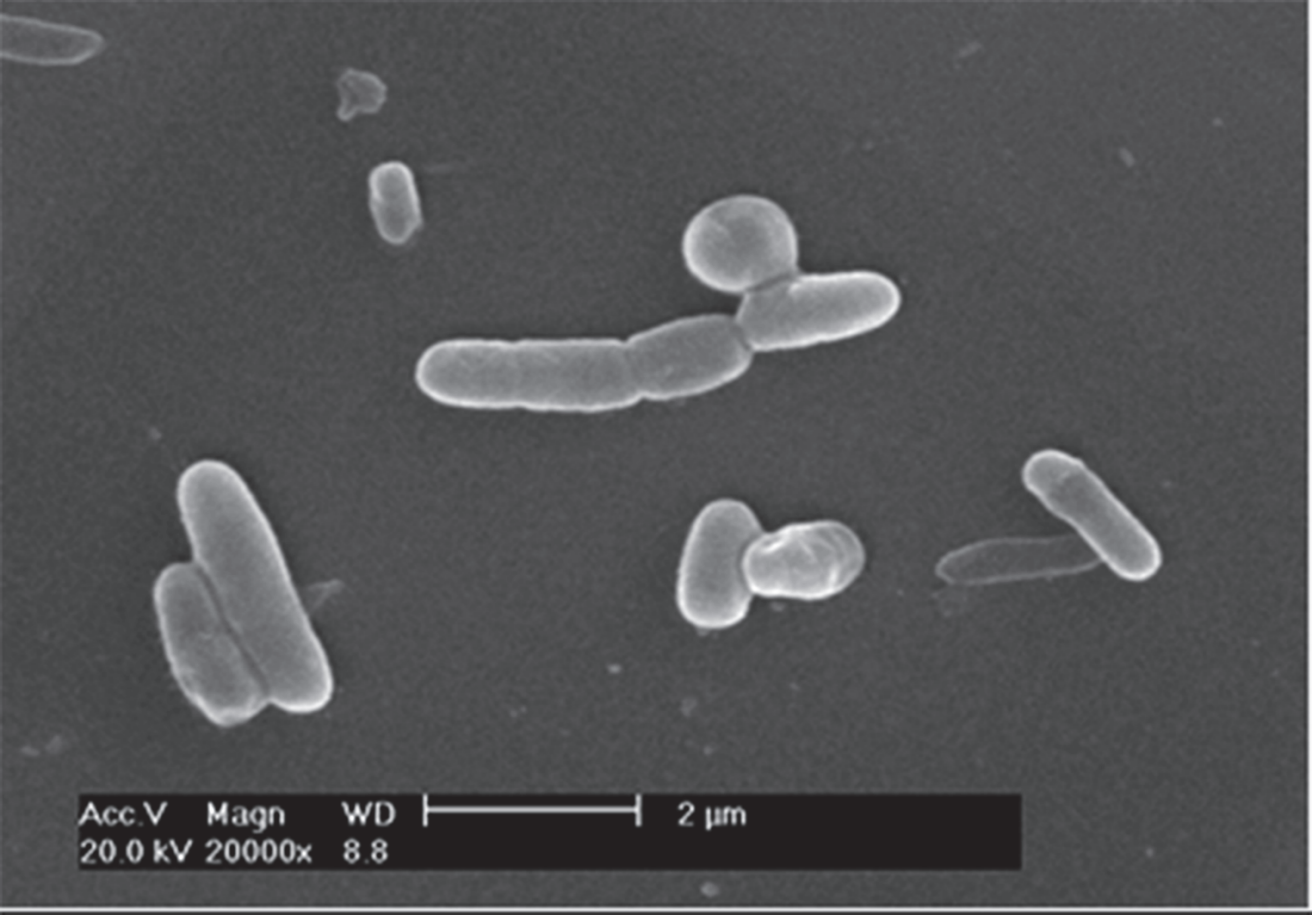
Scanning electron microscrope image of Strain B1.

**Figure 2:**
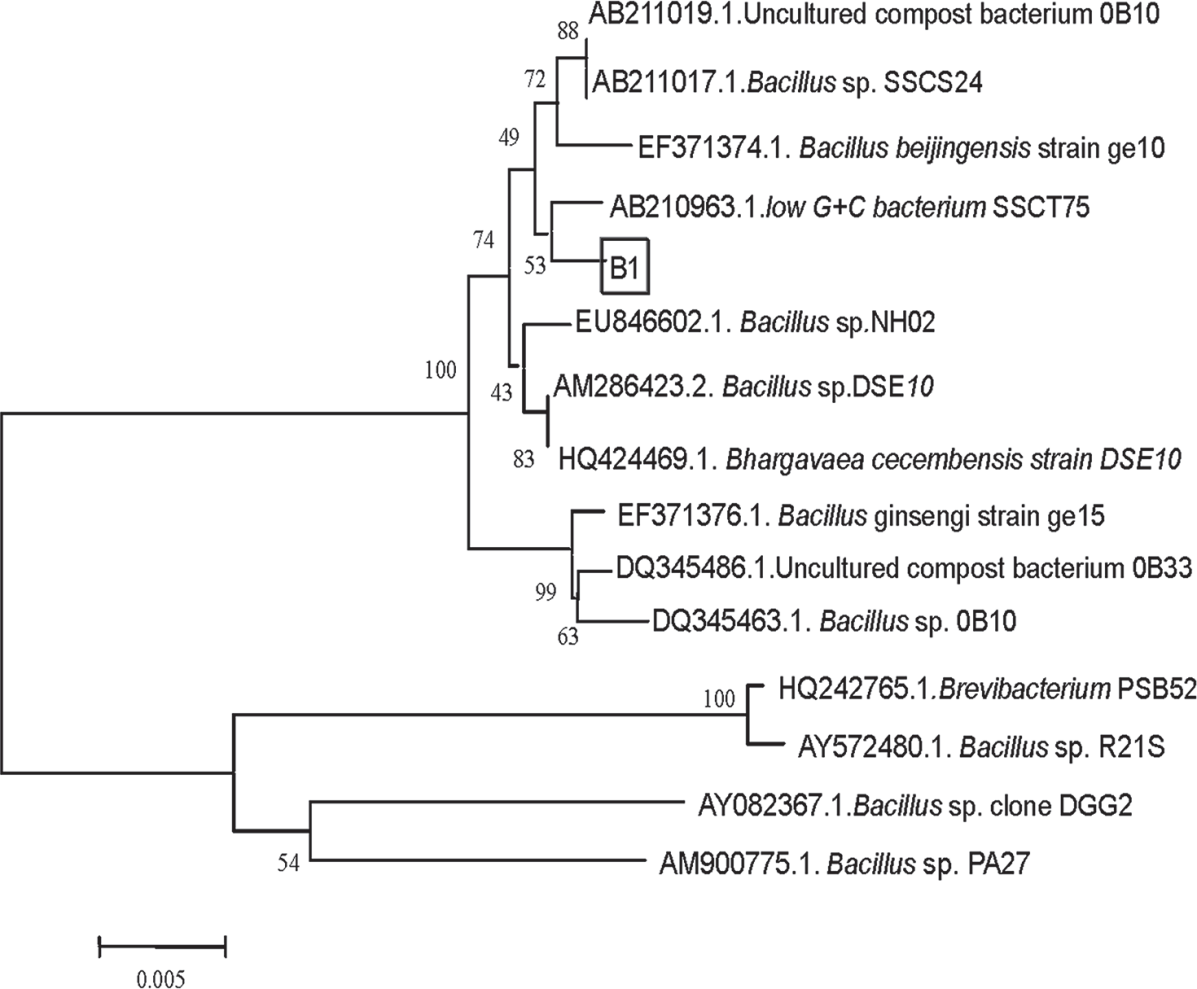
Phylogenetic tree based on 16S rDNA gene of strain B1. The tree was inferred using the neighbor-joining method. Bootstrap values were evaluated from 1000 replications.

The colony of strain B1 was somewhat circular, slightly raised, ivory-white and translucent. Its surface was smooth, moist and shiny. It was gram-positive, rod-shaped (0.7–0.8 μm × 2.0–2.2 μm), without a flagellum, under the scanning electron microscopy (Fig. 1). After sequencing, the 16S rDNA gene of strain B1 (comprising 1542 bp nucleotides) was determined. Comparison of the 16S rDNA gene of strain B1 (GeneBank accession number JN228893) with those available in the GenBank database indicated that strain B1 was most closely related to *Bacillus* SSCT75 (99% homology, accession number AB210963). A phylogenetic tree was constructed based on the 16S rDNA gene of the strain and its closely related sequences in the NCBI database (Fig. 2).

### Toxicity of Bacterial Supernatant to Phytoplankton, Zooplankton and Fish

**Figure 3:**
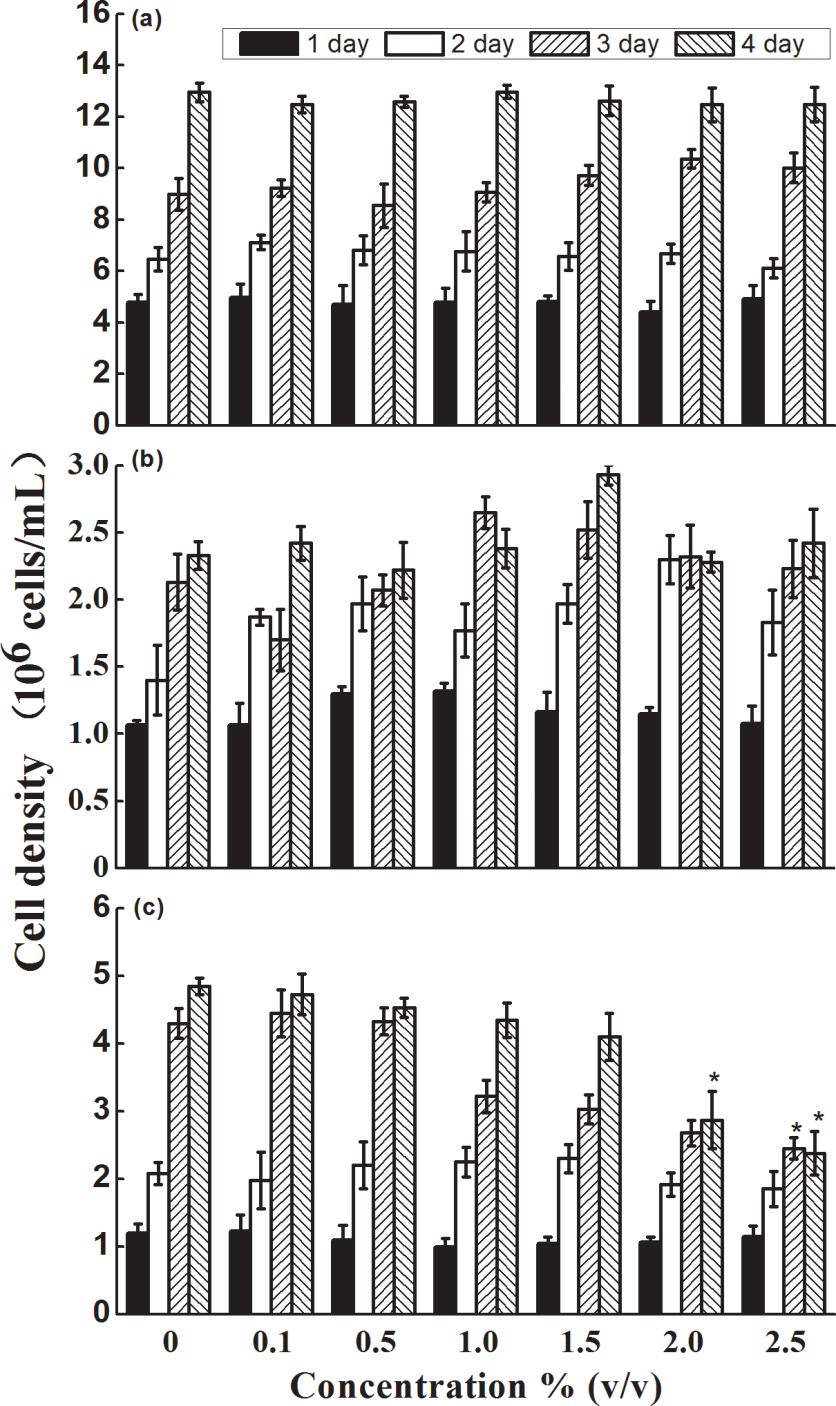
Effects of bacterial supernatant on algal cell densities. (a) *C. vulgaris*, (b) *C. muelleri*, and (c) *I. galbana*. Data represent mean ± standard deviation. Error bars represent standard deviation of triplicate samples. The asterisk (*) indicates a significant difference of p < 0.05 when compared to the control.

For cell densities of *C. vulgaris* and *C. muelleri*, there was no significant difference between treatment and control groups (P > 0.05), and no significant difference between different concentrations of supernatant (P > 0.05). The algicidal rates for *I. galbana* were 2.5, 6.6, 10.3, 15.5, 40.8 and 50.9% after exposure to different concentrations of supernatant for 4 d, respectively (Fig. 3). The LC_50_ values and no observed effect concentration (NOEC) of the bacterial supernatant to *M. mongolica, B. plicatilis* and *P. olivaceus* were shown in Table 1. The supernatant had the highest 24h and 48h LC_50_ values of 8.5 and 5.7% (v/v) to *B. plicatilis*. The 24h and 48h LC_50_ of the bacterial supernatant for *M. mongolica* were 15.4 and 9.0% (v/v), and for *P. olivaceus* were 14.3 and 12.1% (v/v), respectively.

**Table 1:** Acute toxicity of bacterial supernatant to *M. mongolica*, *B. plicatilis* and *P. olivaceus*.

	***M. mongolica***	***B. plicatilis***	***P. olivaceus***
24h LC_50_	15.4 ± 0.2%	8.5 ± 0.3%	14.3 ± 0.3%
48h LC_50_	9.0 ± 0.1%	5.7 ± 0.3%	12.1 ± 0.1%
NOEC	2.0 ± 0.2%	1.0 ± 0.1%	4.0 ± 0.2%

LC_50_, lethal concentration for 50% of organisms; NOEC, no observed effect concentration.

### Purification and Identification of Algicidal Compounds

**Figure 4:**
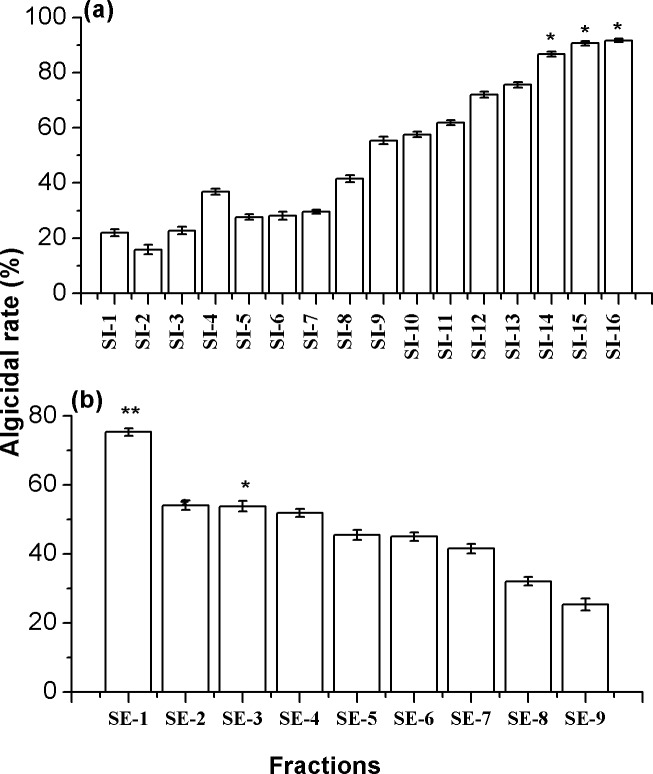
The algicidal activities of fractions against *P. globosa*. (a) fractions eluted from silica gel column; (b) fractions eluted from Sephadex G-15 column. Data represent mean ± standard deviation. Error bars represent standard deviation of triplicate samples. The asterisk (*) indicates a significant difference of p < 0.05, (**) indicates a significant difference of p < 0.01 when compared to the control.

**Figure 5:**
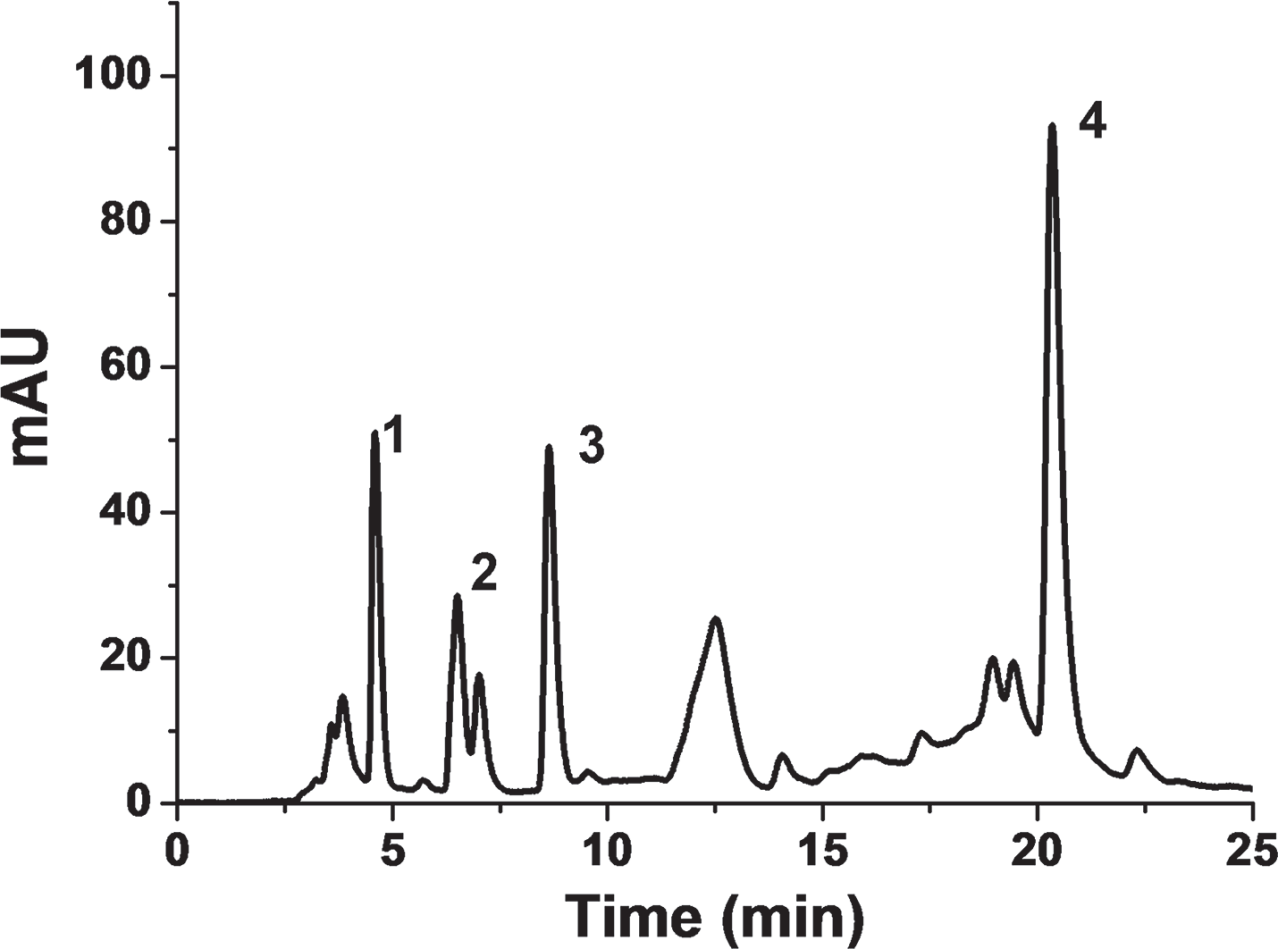
HPLC chromatogram of the fraction SE-1 obtained from Sephadex G-15 column. Chromatographic conditions: column type, 5C_18_-MS-Ⅱ; column size, 4.6 × 250 mm; mobile phase, 0-5-25 min: methanol/water (1/19, v/v)-methanol/water (2/3, v/v)- ethanol/water (2/3, v/v); flow rate, 1 mL/min; detection wavelength, 254 nm; injection volume, 5 μL.

The active fractions SI-14, SI-15 and SI-16 eluted with pure methanol from silica gel column chromatography, which have similar algicidal components (S1 Fig.), were mixed and further isolated by Sephadex G-15 column chromatography (Fig. 4A). Nine fractions were obtained from Sephadex G-15 column and the algicidal activity assays showed that the fraction SE-1 exhibited the strongest algicidal activity against *P. globosa* (Fig. 4B). Fraction SE-1 was then further purified by HPLC and four obvious peaks were obtained (Fig. 5). The algicidal rates of compounds corresponding to peaks 1–4 were 59, 15, 54 and 12%, respectively. The compounds corresponding to peak 1 and 3 were found to have algicidal effect on *P. globosa* and selected for further identification.

**Figure 6:**
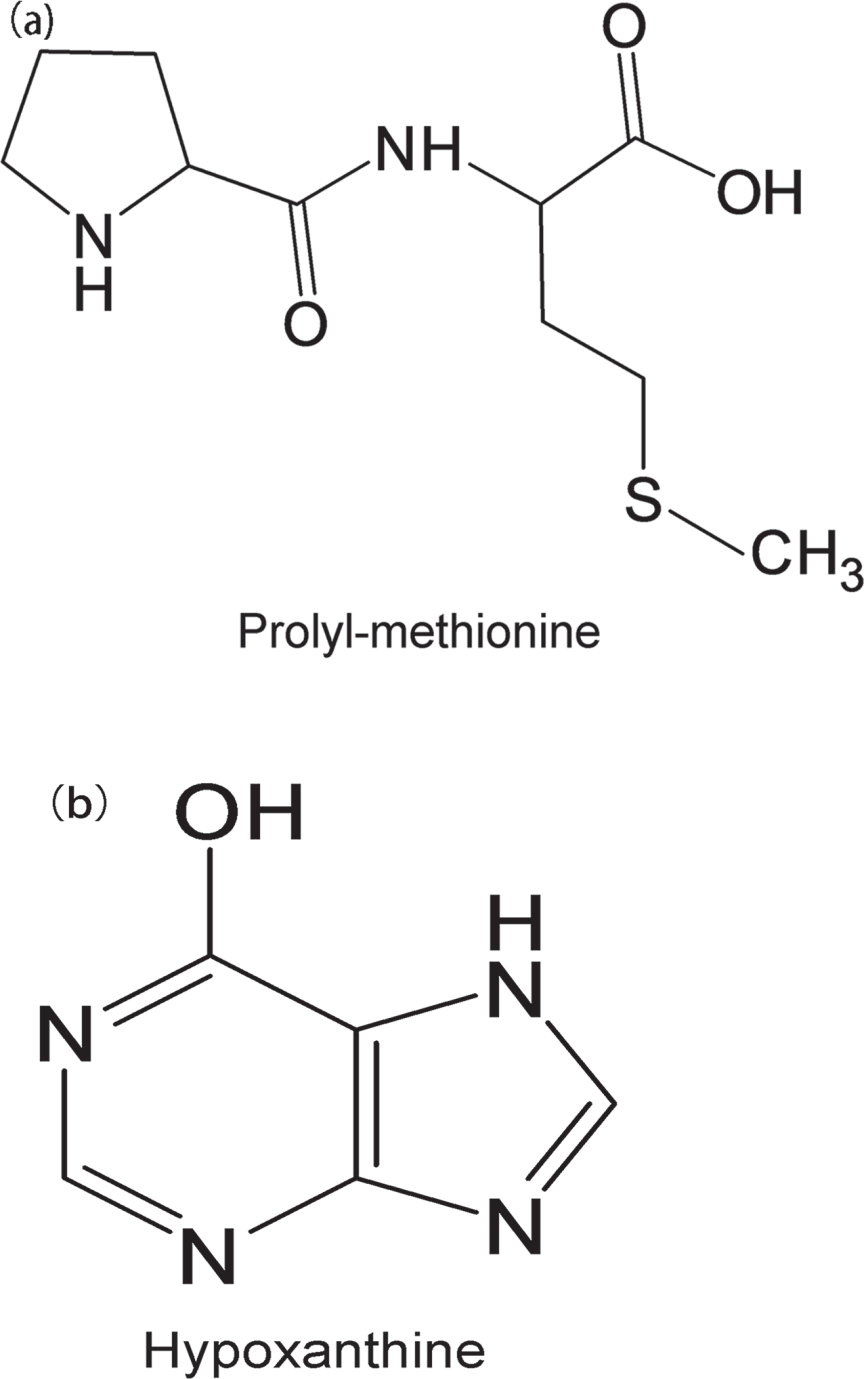
Molecular structures of algicidal compounds corresponding to peak 1 and 3.

Chemical structures of the algicidal compounds corresponding to peaks 1 and 3 were further analyzed by Q-TOF-MS and PeakView Software. Formula Finder of PeakView Software indicated that peak 1 was prolyl-methionine based on the accurate mass information, the isotopic pattern and MS-MS spectra (Table 2). Peak 1 was further confirmed to be prolyl-methionine by Fragments Pane of PeakView Software matching the experimental MS-MS fragments with prolyl-methionine theoretical MS-MS fragments (Table 3, Fig. 6A). Similarly, by the same analytic methods, peak 3 was identified to be hypoxanthine (Tables 1 and 3, Fig. 6B).

**Table 2:** Predicted formula, theoretical mass, experimental mass and mass errors of the molecular ions of the two purified compounds.

**Compounds**	**Predicted formula**	**Polarity type**	**Theoretical m/z**	**Experimental m/z**	**Error (ppm)**
Peak 1	C_10_H_18_N_2_O_3_S	+H	247.1111	247,1112	0.3
Peak 3	C_5_H_4_N_4_O	+H	137.0458	137.0459	−1.0

**Table 3:** Comparison of the experimental MS-MS fragments with the theoretical MS-MS fragments by Fragments Pane of PeakView Software.

**Sample**	**Experimental Mass/Charge (Da)**	**Intensity (%)**	**Error (Da)**
a: Peak 1 (Prolyl-methionine)	70.0655	100.00	0.000
	247.1115	20.37	0.000
b: Peak 3 (Hypoxanthine)	55.0305	23.20	0.001
	65.0136	6.58	0.000
	67.0291	9.34	0.000
	82.0395	21.79	0.013
	92.0243	9.81	0.000
	94.0394	18.27	0.001
	110.0351	49.28	0.000
	119.0349	64.28	0.000
	120.0198	7.62	0.001
	137.0458	100.00	0.000

### Algicidal Mechanisms of the Purified Compounds

**Figure 7:**
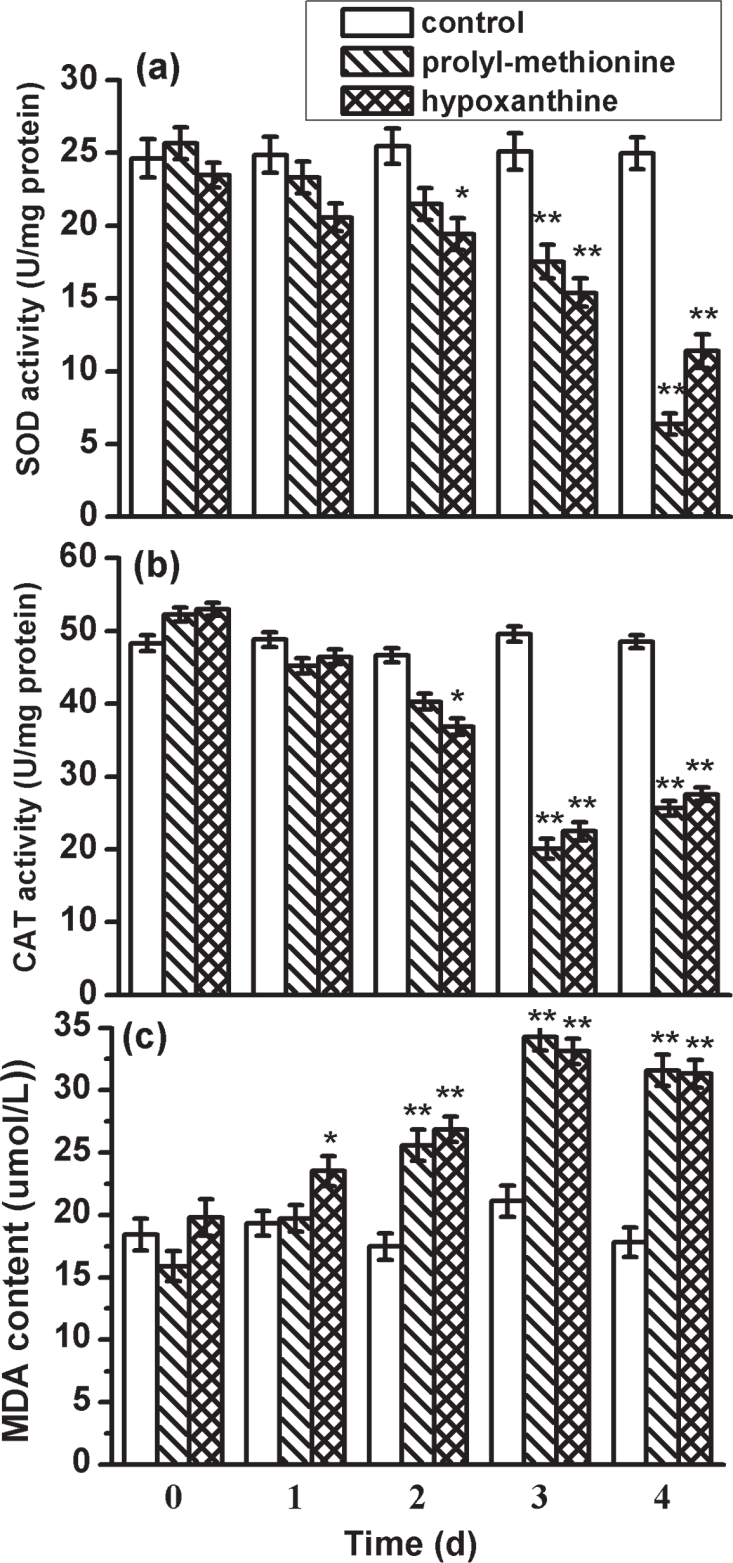
Effects of algicidal compounds on (a) SOD activity, (b) CAT activity and (c) MDA content of *P. globosa*. Data represent mean ± standard deviation. Error bars represent standard deviation of triplicate samples. The asterisk (*) indicates a significant difference of p < 0.05, (**) indicates a significant difference of p < 0.01 when compared to the control

Cellular enzymes activities including SOD and CAT were determined to investigate the cellular defense response induced by prolyl-methionine and hypoxanthine. Meanwhile, MDA content was surveyed to evaluate the damage level of the cell membrane. After the cells were co-cultured with 20 μg/mL prolyl-methionine and hypoxanthine for 4 d, SOD activities of *P. globosa* declined by 75 and 51%, CAT activities decreased from 52.3 to 25.6 U/mg·protein and 52.9 to 27.6 U/mg·protein, respectively (Fig. 7A and 7B). MDA levels in treatment groups were higher than control groups. The MDA concentration of *P. globosa* increased from 15.9 to 31.6 μmol/L and 19.8 to 31.3 μmol/L after 4 d treatment with 20 μg/mL prolyl-methionine and hypoxanthine, respectively (Fig. 7C).

### Acute Toxicities of Purified Compounds to *M. mongolica*

**Figure 8:**
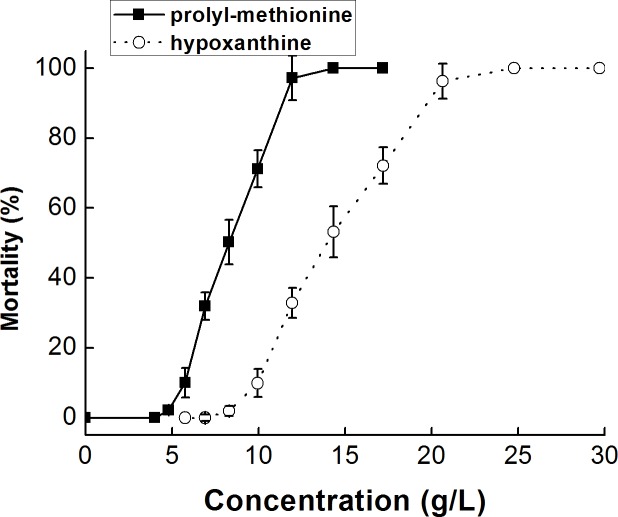
Mortalities of *M. mongolica* treated with different concentrations of purified compounds for 24 h.

The acute toxicities of the two purified compounds were tested to *M. mongolica*. As for *M. mongolica*, the survival rate for 24 h was 100% when the concentration of prolyl-methionine was 4.0 g/L or lower and that of hypoxanthine 5.8 g/L. At 6.9, 8.3, 10.0, 11.9, 14.3 and 17.2 g/L of prolyl-methionine and 10.0, 11.9, 14.3, 17.2 20.6, 24.8 and 29.7 of hypoxanthine, the mortality showed significant differences when compared with the control group (P < 0.01, Fig. 8). In the acute toxicity assessment with prolyl-methionine and hypoxanthine, the 24h LC_50_ values to *M. mongolica* were 7.0 and 13.8 g/L, respectively.

## Discussion

Algicidal bacteria are considered to be key biological controllers in the termination of HABs [17, 29]. *Bacillus* sp. is one of bacterial species exhibiting algicidal ability [30, 31]. An algicidal bacterium against *P. globosa*, strain B1, was obtained in our study. The strain was identified as the genus *Bacillus* based on 16S rDNA gene sequence analysis. Shao et al. [32] isolated a *Bacillus* strain from Lake Donghu with highly lytic efficiency on *Microcystis aeruginosa*. Alamri and Mohamed [33] also indicated the bacterial strain SSZ01 belonging to genus *Bacillus flexus*, which can inhibit the growth of cyanobacteria. However, few studies demonstrated a bacterial strain belonging to genus *Bacillus* which could inhibit the growth of *P. globosa*.

The active algicidal compounds isolated from *Bacillus* sp. B1 cultures were identified as Prolyl-methionine and Hypoxanthine. Prolyl-methionine is a dipeptide which synthesized by dehydration of proline and methionine. Peptide is a well-known anti-algal substance secreted by algicidal bacteria. Jeong et al. [34] extracted a peptide from the culture of *Bacillus* sp. SY-1 which exhibited algicidal activity against *Cochlodinium polykrikoides*. Banin et al. [35] indicated that the coral-bleaching bacterium *Vibrio shiloi* biosynthesized and secreted an extracellular peptide, which inhibited photosynthesis of *Zooxanthellae*. Hypoxanthine is widely used in food industry as an index for evaluating meat or fish freshness [36]. Hypoxanthine is also commonly used in medical and clinical conditions [37, 38]. However, few studies about hypoxanthine on the algicidal activity against harmful algae have been reported. The present is the first to report that hypoxanthine has algicidal activity against *P. globosa*. The finding can increase our knowledge about algicidal substances excreted by marine bacteria.

SOD and CAT are two important antioxidant enzymes in cells that protect organisms against damages caused by H_2_O_2_ and O_2_^−^ [39]. MDA is an indicator of the lipid peroxidation under stress conditions [40]. In this study, the SOD and CAT activities of *P. globosa* cells were decreased under prolyl-methionine or hypoxanthine stress after 4 d of cultivation. Meanwhile, the MDA levels of treatment groups were significantly higher than those of the control groups. This observation demonstrated the decrease of antioxidant capacity of *P. globosa* after exposed to algicidal compounds led to more serious oxidative damage, which further resulted in the increase of MDA contents owing to keeping the normal function of membranes. The results indicated that prolyl-methionine and hypoxanthine exhibited algicidal activity against *P. globosa* by destroying the cell antioxidant systems. Luo et al. [6] identified a type of triterpenoid saponin from *Streptomyces* sp. L74, which could disrupt the antioxidant systems of *Microcystis aeruginosa* cells. Zhang et al. [41] also indicated that the culture supernatant of *Brevibacterium* sp. BS01 inhibited the growth of *Alexandrium tamarense* by disrupting the antioxidant systems of algal cells.

Toxicity to marine organisms is one of the most important factors that influence the use of algicides [42, 43]. Phytoplankton plays an important role in ecosystem balance as the primary producer. The algae of *C. vulgaris, C. mulleri* and *I. galbana* were commonly distributed in the marine system. *M. mongolica* and *B. plicatilis* have been widely used in ecotoxicological studies because of their convenient cultivation, short life cycle and high sensitivity to many toxicants [44]. *P. olivaceus* was chosen to evaluate the ecological safety of algicides because of its importance as an economic aquatic animal and major aquaculture organism. The toxicity test indicated that the supernatant of strain B1 showed weak algicidal activity against *C. vulgaris, C. muelleri* and *I. galbana* at the concentration of 1.0% (v/v). The 48h LC_50_ values of the supernatant to *M. mongolica daday, B. plicatilis* and *P. olivaceus* were 9.0, 5.7 and 12.1% (v/v), which were 9.0, 5.7 and 12.1 times higher than that to *P. globosa* (1.0%, v/v), respectively. In order to evaluate the toxicity of purified compounds, acute toxicities of rolyl-methionine and hypoxanthine against *M. mongolica* were tested. Results indicated that *M. mongolica* could easily survive within the concentration of 4.0 and 5.8 g/L, respectively. Zhou et al. [44] indicated the 24h LC_50_ of garlic solution to *M. mongolica* and *B. plicatilis* were 0.33 and 0.17% (v/v), respectively. Kim et al. [45] reported the 72h LC_50_ of thiazolidinedione derivatives TD49 to *P. olivaceus* was 0.58 μmol/L. Therefore, the algicidal substances of bacterium *Bacillus* sp. B1 can be applied as ecologically biocontrol agents against the blooms of *P. globosa*.

In summary, our results showed that *Bacillus* sp. B1 inhibited the growth of *P. globosa* by excreting active substances, which were biologically safe for other marine organisms. Therefore, the development of specific and environment-friendly to derivatives from rolyl-methionine and hypoxanthine for use in the control of HABs is possible. The synergistic effect of rolyl-methionine and hypoxanthine for controlling *P. globosa* should be further study.

## Supporting Information

S1 Fig Ultraviolet scanning spectrogram of fractions SI-14, SI-15 and SI-16. (DOC)

## References

[1] Paerl HW, Huisman J (2008) Blooms like it hot. Science 320: 57–58. 18388279 10.1126/science.1155398

[2] Pierce RH, Henry MS (2008) Harmful algal toxins of the Florida red tide (*Karenia brevis*): natural chemical stressors in South Floria coastal ecosystems. Ecotoxicology 17(7): 623–631. 10.1007/s10646-008-0241-x 18758951 PMC2683401

[3] Xavier M, Gregory JD (2002) Microbial community interactions and population dynamics of an algicidal bacterium active against *Karenia brevis* (Dinophyceae). Harmful algae 1(12): 277–293.

[4] Anderson DM (1997) Turing back the harmful red tide-commentary. Nature 388: 513–514.

[5] Su JQ, Yang XR, Zheng TL, Tian Y, Jiao NZ et al. (2007) Isolation and characterization of a marine algicidal bacterium against the toxic dinoflagellate *Alexandrium tamarense* . Harmful Algae 6: 799–810. 10.1007/s12275-008-0141-z 19229486

[6] Luo JF, Wang Y, Tang SS, Liang JW, Lin WT et al. (2013) Isolation and identification of algicidal compound from *Streptomyces* and algicidal mechanism to *Microcystis aeruginosa* . PLoS ONE 8(10): 1–14. 10.1371/journal.pone.0076444 24098501 PMC3789699

[7] Chen WM, Sheu FS, Sheu SY (2011) Novel L-amino acid oxidase with algicidal activity against toxic cyanobacterium *Microcystis aeruginosa* synthesized by a bacterium *Aquimarina* sp. Enzyme Microb. Tech. 49: 372–379. 10.1016/j.enzmictec.2011.06.016 22112563

[8] Wang BX, Zhou YY, Bai SJ, Su JQ, Tian Y et al. (2010) A novel marine bacterium algicidal to the toxic dinoflagellate *Alexandrium tamarense* . Lett. Appl. Microbiol. 51: 552–557. 10.1111/j.1472-765X.2010.02936.x 20880149

[9] Somdee T, Sumalai N, Somdee A (2013) A novel actinomycete *Streptomyces aurantiogriseus* with algicidal activity against the toxic cyanobacterium *Microcystis aeruginosa* . J. Appl. Phycol. 25: 1587–1594.

[10] Lovejoy C, Bowman JP, Hallegraeff GM (1998) Algicidal effects of a novel marine *Pseudoalteromonas* isolate (Class *Proteobacteria*, Gamma subdivision) on harmful algal bloom species of genera *Chattonella, Gymnodinium*, and *Heterosigma* . Appl. Environ. Microbiol. 64: 2806–2813. 9687434 10.1128/aem.64.8.2806-2813.1998 PMC106776

[11] Azam F (1998) Microbial control of oceanic carbon flux: the plot thickens. Science 280: 694–696.

[12] Cho JY (2012) Algicidal activity of marine *Alteromonas* sp. KNS-16 and isolation of active compounds. Biosci. Biotechnol. Biochem. 76: 1452–1458. 22878186 10.1271/bbb.120102

[13] Lee SO, Kato J, Nakashima K, Kuroda A, Ikeda T et al. (2002) Cloning and characterization of extracellular metal protease gene of the algicidal marine bacterium *Pseudoalteromonas* sp. strain A28. Biosci. Biotechnol. Biochem. 66: 1366–1369. 12162559 10.1271/bbb.66.1366

[14] Imamura N, Motoike I, Noda M, Adachi K, Konno A et al. (2000) Argimicin A, a novel anti-cyanobacterial substance produced by an algae-lysing bacterium. J. Antibiot. 53: 1317–1319. 11213296 10.7164/antibiotics.53.1317

[15] Ahn CY, Joung SH, Jeon JW, Kim HS, Yoon BD et al. (2003) Selective control of cyanobacteria by surfactin-containing culture broth of *Bacillus subtilis* C1. Biotechnol. Lett. 25: 1137–1142. 12967000 10.1023/a:1024508927361

[16] Wang XL, Gong LY, Liang SK, Han XR, Zhu CJ et al. (2005) Algicidal activity of rhamnolipid biosurfactants produced by *Pseudomonas aeruginosa* . Harmful Algae 4:433–443.

[17] Yoshikawa K, Adachi K, Nishijima M, Takadera T, Tamaki S et al. (2000) β-Cyanoalanine production by marine bacteria on cyanide-free medium and its specific inhibitory activity toward cyanobacteria. Appl. Environ. Microbiol. 66(2): 718–722. 10653741 10.1128/aem.66.2.718-722.2000 PMC91886

[18] Nakashima T, Miyazaki Y, Matsuyama Y, Muraoka W, Yamaguchi K et al. (2006) Producing mechanism of an algicidal substance against red tide phytoplankton in a marine bacterium γ-*proteobacterium* . Appl. Microbiol. Biot. 73:684–690. 16850298 10.1007/s00253-006-0507-2

[19] Shao JH, Li RH, Lepo JE, Gu JD (2013) Potential for control of harmful cyanobacterial blooms using biologically derived substances: Problems and prospects. J. Environ. Manage. 125: 149–155. 10.1016/j.jenvman.2013.04.001 23660535

[20] Blauw AN, Los FJ, Huisman J, Peperzak L (2010) Nuisance foam events and *Phaeocystis globosa* blooms in Dutch coastal waters analyzed with fuzzy logic. J. Marine Syst. 83: 115–126.

[21] Hai X, Paerl HW, Qin BQ, Zhu GW, Gao G (2010) Nitrogen and phosphorus inputs control phytoplankton growth in eutrophic Lake Taihu, China. Limnol. Oceanogr. 55(1): 420–432.

[22] Zheng XW, Zhang BZ, Zhang JL, Huang LP, Lin J et al. (2013) A marine algicidal actinomycete and its active substance against the harmful algal bloom species *Phaeocystis globosa* . Appl. Microbiol. Biot. 97: 9207–9215. 10.1007/s00253-012-4617-8 23224407

[23] Galtier N, Gouy M, Gautier C (1996) Two graphic tools for sequence alignment and molecular phylogeny. Comput. Appl. Biosci. 12(6): 543–548 9021275 10.1093/bioinformatics/12.6.543

[24] Saitou N, Nei M (1987) The neighbor-joining method: a new method for reconstructing phylogenetic trees. Mol. Biol. Evol. 4(4): 406–25. 3447015 10.1093/oxfordjournals.molbev.a040454

[25] Beauchamp C, Frodovich I (1971) Superoxide dismutase: improved assays and an assays applicable acrylamide gels. Anal. Biochem. 44: 276–287. 4943714 10.1016/0003-2697(71)90370-8

[26] Li HS (2006) Experimental principles and techniques of plant physiology and biochemistry. Higher Education, Beijing, China.

[27] Uchimaya M, Mihara M (1978) Determination of malonaldehyde precursor in tissues by thiobarbituric acid test. Anal. Biochem. 86: 271–278. 655387 10.1016/0003-2697(78)90342-1

[28] Schoemann V, Becquevort S, Stefels J, Rousseau W, Lancelot C (2005) *Phaeocystis* blooms in the global ocean and their controlling mechanisms: a review. J. Sea Res. 53: 43–66.

[29] Van RM, Janse I, Noordkamp DJB, Gieskes WWC (2000) An inventory of factors that affect polysaccharide production by *Phaeocystis globosa* . J. Sea Res. 43: 297–306.

[30] Yang L, Wei HY, Masaharu K, Kenichi I, Lin JS et al. (2012) Isolation and characterization of bacterial isolates algicidal against a harmful bloom-forming cyanobacterium *Microcystis aeruginosa* . Biocontrol Sci. 17: 107–114. 23007101 10.4265/bio.17.107

[31] Kim MJ, Yun SY, Lee SJ (2008) Isolation, identification, and algicidal activity of marine bacteria against *Cochlodinium polykrikoides* . J. Appl. Phycol. 20: 1069–1078.

[32] Shao J, Jiang Y, Wang Z, Peng L, Luo S et al. (2014) Interactions between algicidal bacteria and the cyanobacterium *Microcystis aeruginosa*: lytic characteristics and physiological responses in the cyanobacteria. Int. J. Environ. Sci. Technol. 11: 469–476.

[33] Jancula D, Suchomelova J, Gregor J, Smutna M, Marsalek B (2007) Effects of aqueous extracts from five species of the family *Papaveraceae* on selected aquatic organisms. Environ. Toxicol. 22: 480–486. 17696132 10.1002/tox.20290

[34] Jeong SY, Ishida K, Ito Y, Okada S, Murakami M (2003) Bacillamide, a novel algicide from the marine bacterium, *Bacillus* sp. SY-1, against the harmful dinoflagellate, *Cochlodinium polykrikoides* . Tetrahedron Lett. 44: 8005–8007.

[35] Banin E, Khare SK, Naider F, Rosenberg A (2001) Proline-Rich peptide from the coral pathogen *Vibrio Shiloi* that inhibits photosynthesis of *Zooxanthellae* . Appl. Environ. Microbiol. 64(4): 1536–1541. 11282602 10.1128/AEM.67.4.1536-1541.2001 PMC92766

[36] Balladin DA, Natinesingh D, Stoute VA, Ngo ATT (1997) Immobilization of xanthine oxidase and its use in the quantitation of hypoxanthine in fish muscle tissue extracts using a flow injection method. Appl. Biochem. Biotech. 62: 317–328.

[37] Farthing D, Sica D, Gehr T, Wilson B, Fakhry I et al. (2007) An HPLC method for determination of inosine and hypoxanthine in human plasma from healthy volunteers and patients presenting with potential acute cardiac ischemia. J. Chromatogr. B 854: 158–164. 17466604 10.1016/j.jchromb.2007.04.013

[38] Farthing DE, Sica D, Hindle M, Edinboro L, Xi L et al. (2011) A rapid and simple chemiluminescence method for screening levels of inosine and hypoxanthine in non-traumatic chest pain patients. Luminescence 26: 65–75. 10.1002/bio.1187 20017127

[39] Valentine JS, Wertz DL, Lyons TJ, Liou LL, Goto JJ et al. (1998) The dark side of dioxygen biochemistry. Curr. Opin. Chem. Biol. 2(2): 253–62. 9667937 10.1016/s1367-5931(98)80067-7

[40] Gillespie KM, Rogers A, Ainsworth EA (2010) Growth at elevated ozone or elevated carbon dioxide concentration alters antioxidant capacity and response to acute oxidative stress in soybean (Glycine max). J. Exp. Bot. 62: 2667–267. 21282325 10.1093/jxb/erq435

[41] Zhang HJ, An XL, Zhou YY, Zhang BZ, Zhang S et al. (2013) Effect of oxidative stress induced by *Brevibacterium* sp. BS01 on a HAB causing species-*Alexandrium tamarense* . PLoS ONE 8(5): 1–9. 10.1371/journal.pone.0063018 23667564 PMC3648478

[42] Alamri SA, Mohamed ZA (2013) Selective inhibition of toxic cyanobacteria by b-carboline-containing bacterium *Bacillus flexu*s isolated from Saudi freshwaters. Saudi J. Bio. Sci. 20: 357–363. 10.1016/j.sjbs.2013.04.002 24235872 PMC3824139

[43] Vanhaecke P, Persoone G., Claus C, Sorgeloos P (1981) Proposal for a short-term toxicity test with *Artemia nauplii* . Ecotoxi. Environ. Safe. 5: 382–387. 7297475 10.1016/0147-6513(81)90012-9

[44] Zhou LH, Chen XH, Zheng TL (2010) Study on the ecological safety of algacides: a comprehensive strategy for their screening. J. Appl. Phycol. 22: 803–811.

[45] Kim SJ, Yim EC, Park IT, Kim SW, Cho H (2011) Comparison of the acute toxicities of novel algicides, thiazolidinedione derivatives TD49 and TD53, to various marine organisms. Environ. Toxicol. Chem. 30: 2810–2816 10.1002/etc.691 21932297

