# Supplementary figures and images for: Toxic Effect of a Marine Bacterium on Aquatic Organisms and Its Algicidal Substances against Phaeocystis globosa
Source: PLoS One. 2015 Feb 3;10(2):e0114933. doi: 10.1371/journal.pone.0114933 (PMC4315471; doi:10.1371/journal.pone.0114933)

**Figure S1**


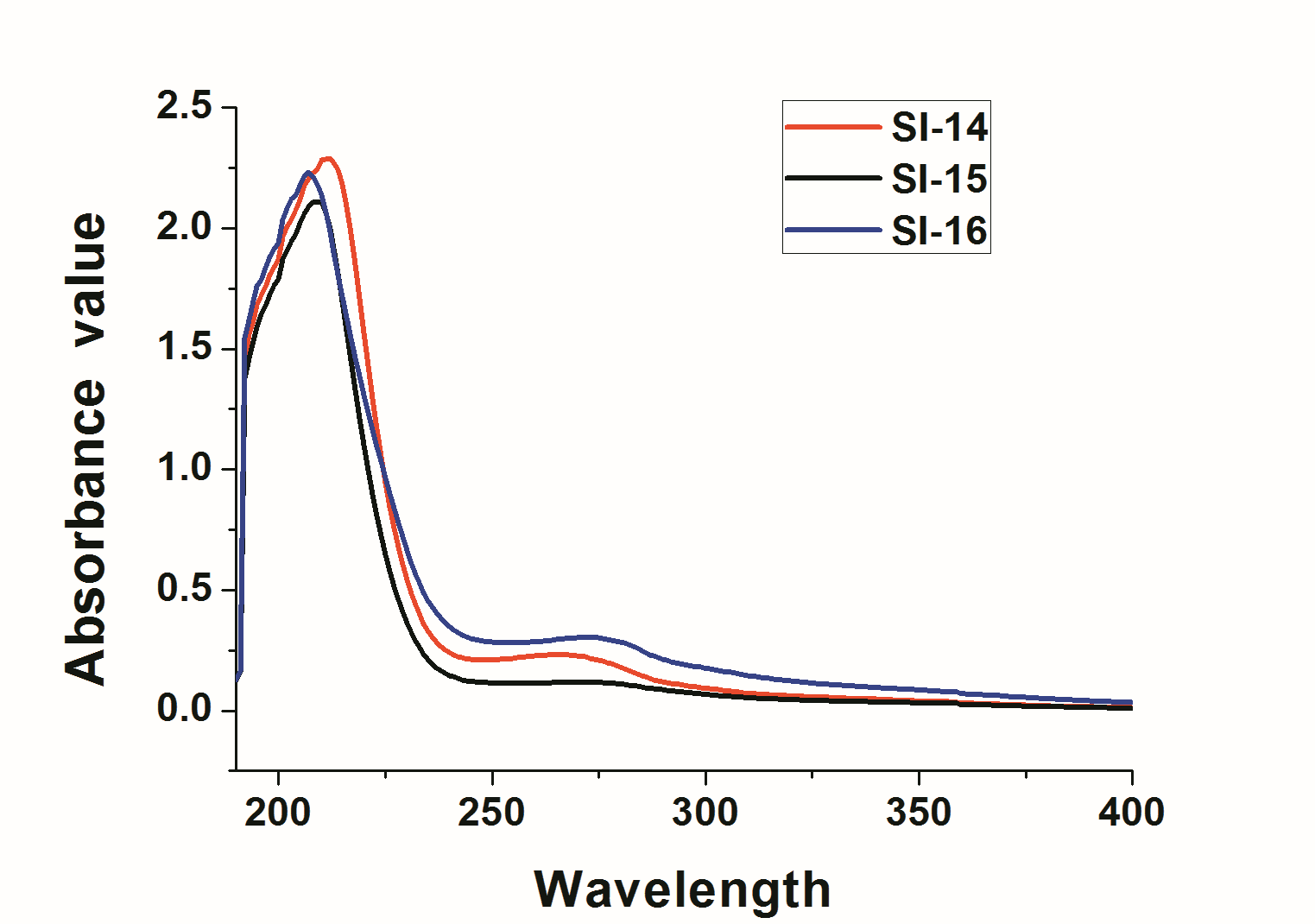

Supplement: S1 Fig — (DOC) [file pone.0114933.s001.doc]
